# The usability of ventilators: a comparative evaluation of use safety and user experience—an editorial response

**DOI:** 10.1186/s13054-017-1751-9

**Published:** 2017-07-06

**Authors:** Edwin Coombs

**Affiliations:** Intensive Care, Draeger, Inc., 3135 Quarry Road, Telford, PA 18969 USA

In response to the recent article in *Critical Care* by Morita et al. [1] entitled “The usability of ventilators: a comparative evaluation of use safety and user experience”, I agree that intensive care unit (ICU) safety and ventilator management are of paramount concern. The need for proper training and clinician education remains a constant priority for many ventilator manufacturers. To that end, the US Food and Drug Administration (FDA) now requires usability testing as part of the 510(k) clearance application [2], and I thank Morita et al. for addressing this important issue.

I would like to point out several discussion topics that I feel result in selection, but unintentional, bias to the study which may have resulted in the outcomes listed in the results of Morita et al. Selection bias occurs when researchers recruit an unrepresentative sample population. The sample population differs in some significant way from the population that generated the sample population [3]. Therefore, generalization of results should be made with caution. The test participants that were recruited were from three hospitals: Duke, Wake-Med, and UNC. These facilities currently utilize the Servo-I, PB840, and Hamilton G5 devices. Having the actual test device or immediate predecessor device of the study device provides test participants entering the testing process with advanced knowledge and experience versus that of equipment that is not used at their respective facilities.

Additionally, the study used “exploration-based training”. According to the FDA guidance on usability tests: “To the extent practicable, the content, format, and method of delivery of training given to test participants should be comparable to the training that actual users would receive”. Respiratory therapists (RTs) would typically receive training from the manufacturer or from manufacturer-certified trainers; therefore, the study should have offered such training before each test session [4]. Knowing that the study did not utilize manufacturer-trained clinical educators, the actual training used as the basis for the study must be questioned.

With these two points explained in the study design, I am of the opinion that the execution of the study with regard to recruited candidates and trainers introduces a level of bias that can easily skew the reported results [5]. I would suggest that a future study be performed with participants that have absolutely no prior experience of the study devices and that training be conducted by a certified manufacturer’s educator.

Thank you again to Morita et al. [1] for posting this study. ICU ventilator safety is of critical importance to everyone who plays a role in the delivery of care.
